# A Case of Diphtheria and Infectious Mononucleosis Co-Infection in a Partially Vaccinated Boy

**DOI:** 10.7759/cureus.11227

**Published:** 2020-10-28

**Authors:** Gladys Cyril, Balram Rathish, Arun Wilson, Anup Warrier, Vineeth Viswam

**Affiliations:** 1 Pediatrics, Aster Medcity, Kochi, IND; 2 Infectious Diseases, Aster Medcity, Kochi, IND; 3 Otolaryngology - Head and Neck Surgery, Aster Medcity, Kochi, IND

**Keywords:** diphtheria, infectious mononucleosis, co-infection, vaccination

## Abstract

We report the case of an eight-year-old partially immunized boy who presented with presumed bacterial tonsillitis. He was initially prescribed amoxicillin-clavulanic acid which resulted in the development of an erythematous maculopapular over the face which spread to the trunk and extremities including palms and soles and resolved over the next three days. He was diagnosed to have diphtheria and infectious mononucleosis (IMN) co-infection. He made an uneventful recovery and an extensive review of the literature showed that the incidence of diphtheria and IMN co-infection is a relatively rare clinical entity. We wish to highlight the possibility of such co-infections which often mimic one another.

## Introduction

Diphtheria is caused by Corynebacterium diphtheria which is an aerobic, gram-positive, non-motile, non-capsulated, non-spore-forming, toxin-producing bacillus. It is a vaccine-preventable disease and the hallmark of the disease involves a pseudomembrane on the site of colonization, usually involving the tonsils [1]. Most cases of infectious mononucleosis (IMN) are caused by the Epstein-Barr virus (EBV). After exposure, the EBV infects the epithelial cells of the salivary glands and the oropharynx. Lymphoid hyperplasia occurs and may present as predominantly cervical lymphadenopathy and tonsillitis, making it a close differential diagnosis for diphtheria [2]. These infections can mimic one another due to the similarity between their clinical presentations. An extensive review of the literature showed that the incidence of diphtheria and IMN co-infection is very rare with only three reported cases [3-5]. We wish to highlight the possibility of such co-infections that can mimic one another and may often be missed.

## Case presentation

**Figure 1:**
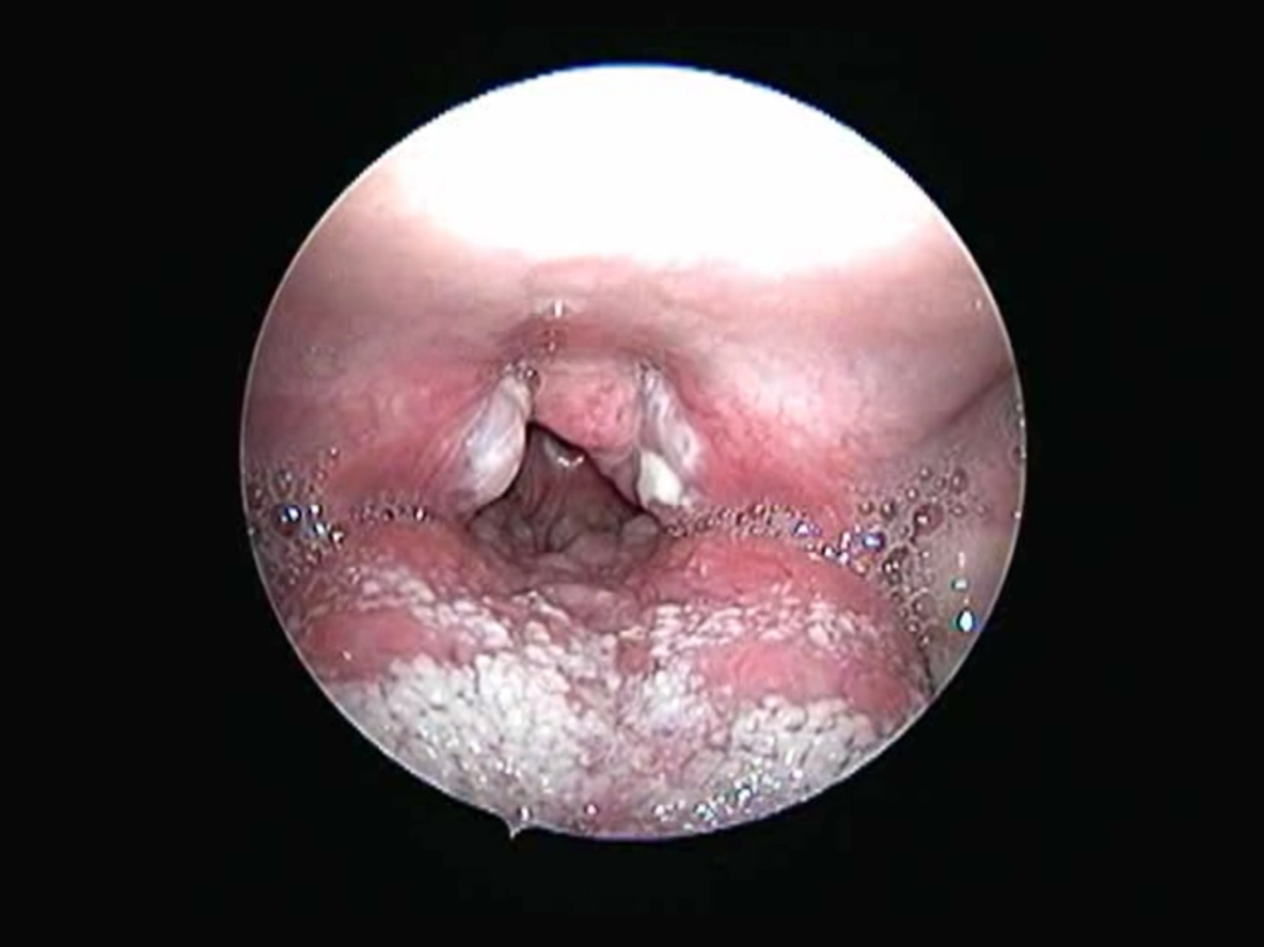
Greyish-white membrane seen over enlarged tonsils.

An eight-year-old boy with no co-morbid illnesses presented with a history of sore throat and swelling of the neck for the past three days. He had been under treatment elsewhere with amoxicillin-clavulanate for presumed bacterial tonsillitis and had been on the antibiotic since the onset of symptoms. His medical history was remarkable for his partially immunized status with missed booster doses of the diphtheria-pertussis-tetanus (DPT) vaccine. On clinical examination, he was febrile with a temperature of 100 F. Examination of the throat showed enlarged tonsils with evidence of a greyish membrane, and neck examination showed cervical lymphadenopathy (Figure 1). Abdominal examination revealed hepatosplenomegaly. Workup showed a total leukocyte count of 27,690 (4000-11,000/uL) with 66% lymphocytes and atypical lymphocytes on the peripheral blood smear. His C-reactive protein (CRP) was 12 (<10 mg/dl), erythrocyte sedimentation rate (ESR) was 16 (0-15 mm/h), aspartate aminotransferase (AST) was 415 (0-35 u/L) and alanine aminotransferase (ALT) was 293 (0-35 u/L).

A throat swab was obtained for culture & sensitivity, and testing for diphtheria. Given his partial immunization status and the clinical picture, he was started on anti-diphtheritic serum (80,000 units) along with parenteral crystalline penicillin and oral Clindamycin. Two days later, he developed blood-stained nasal discharge along with hoarseness of voice and stridor. A lateral radiograph of the neck was normal. A mono spot was positive. An EBV panel consisting of IgM EBV-viral capsid antigen (VCA), IgG-VCA, IgG-early antigen, and nuclear antigen was sent on day five of illness, and IgG early antigen and IgM-VCA came positive. EBV quantitative polymerize chain reaction (PCR) was sent on the same day, which showed significant copies (36,275 copies/ml) confirming primary EBV infection.

His throat swab inoculated on sheep blood, MacConkey, and Tellurite blood agar cultures, but all were sterile. A repeat sample for inoculation on Loeffler's agar could only be collected 36 hours after starting crystalline penicillin and this too was sterile, and an Elek test could not be performed. However, PCR was positive for a toxigenic strain of Corynebacterium diphtheriae, confirming the diagnosis of diphtheria.

**Figure 2:**
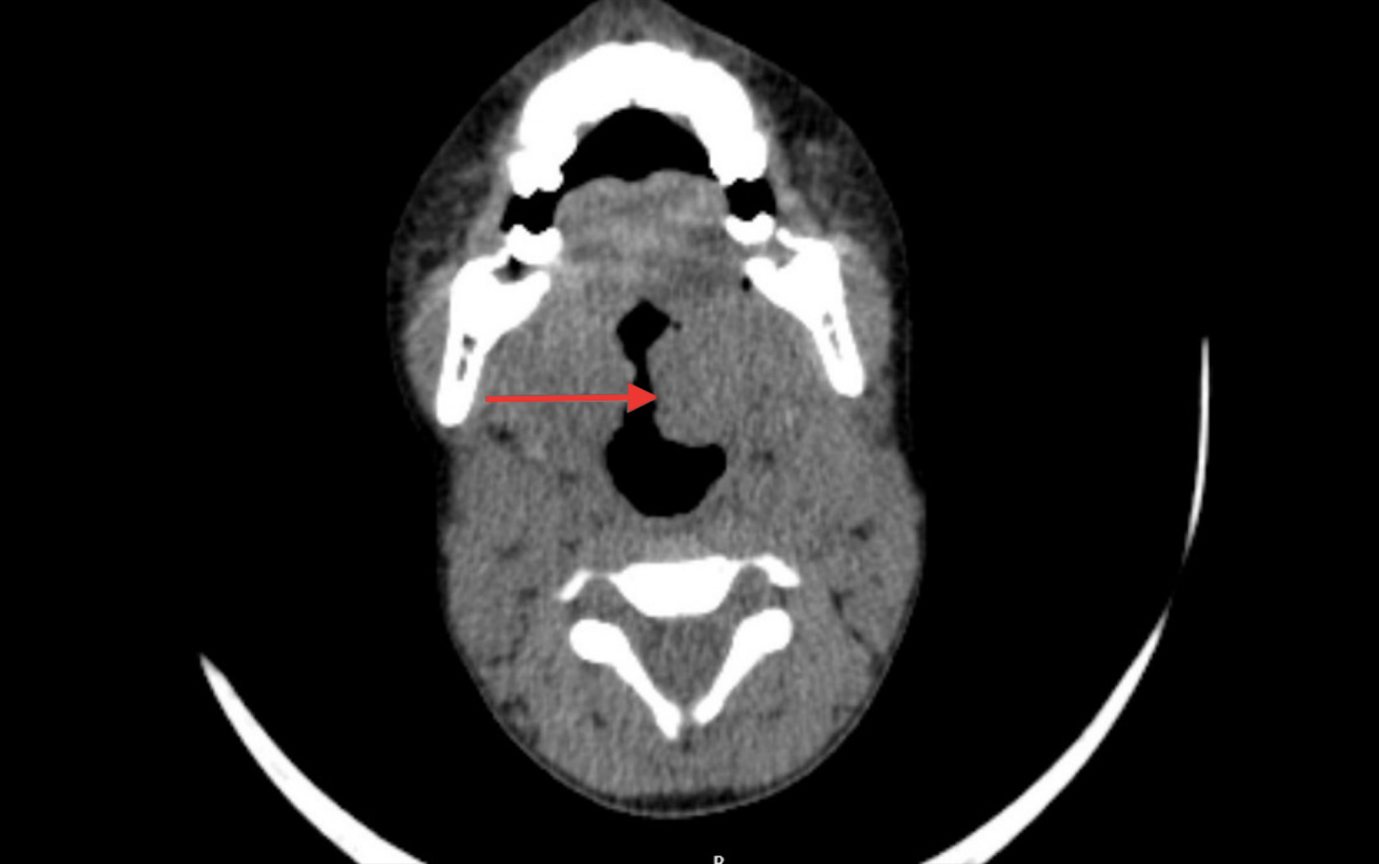
Axial cut of CT showing considerable enlargement of the tonsils (Red arrow).

**Figure 3:**
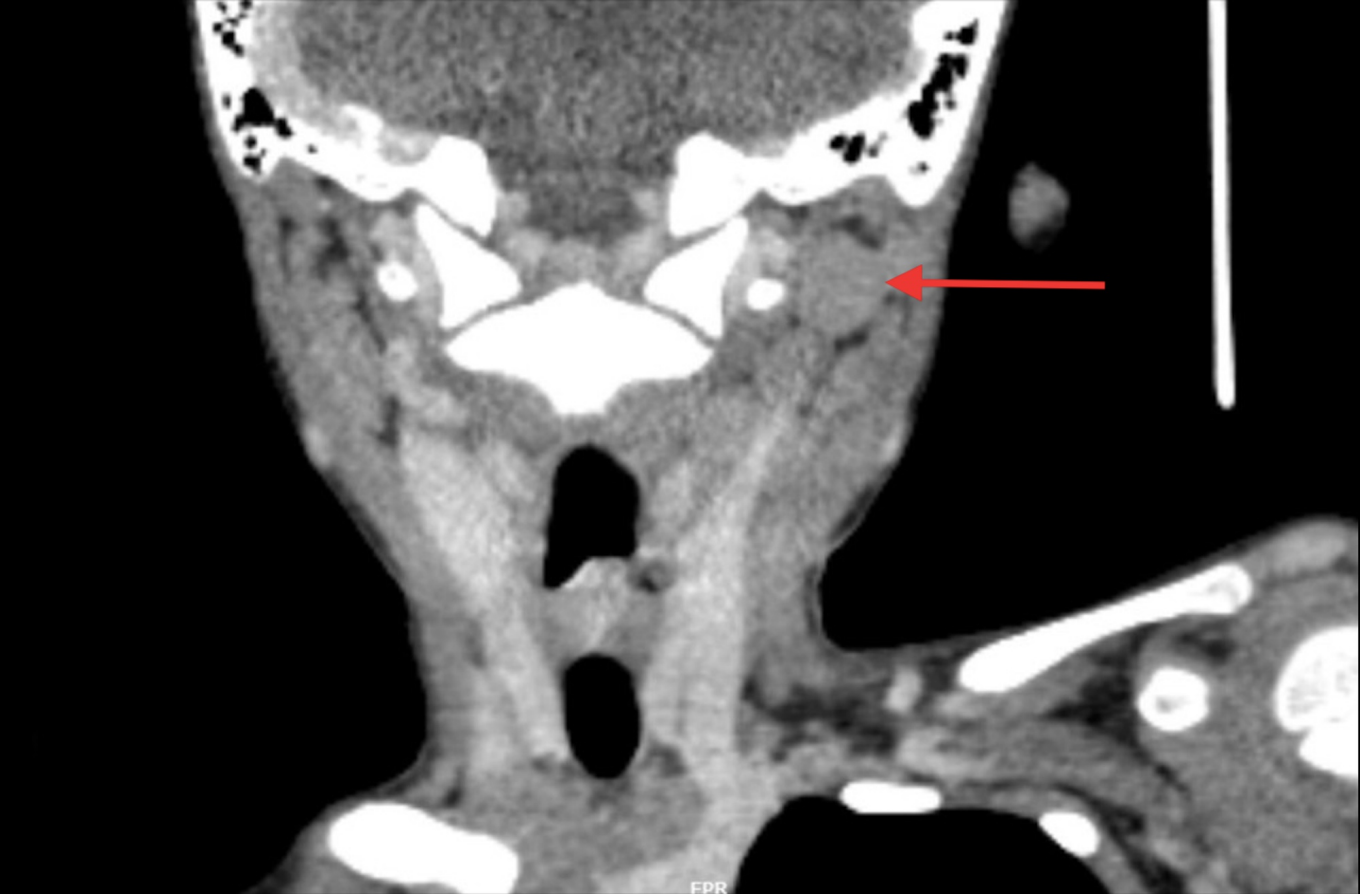
Coronal cut of CT showing multiple enlarged cervical nodes on the left side of the neck (Red arrow).

By day seven of illness, he improved clinically with a decrease in soft tissue swelling, reduction in hoarseness, and disappearance of stridor. However, he continued to spike a fever. In view of the persisting fever spikes despite appropriate antibiotics, the possibility of an abscess or suppurative cervical lymphadenitis was considered. CT scan of the neck was done and it showed considerable enlargement of the tonsils, adenoids, edema of the posterior glottis, and cervical lymphadenopathy (Figures 2, 3). The antibiotics were continued and the fever spikes resolved on day 10 of the illness. 

He developed a rash suggestive of IMN on day 10 of the illness (eight days after receiving amoxicillin-clavulanic acid) which started as an erythematous maculopapular over the face, spreading to the trunk and extremities including palms and soles. This rash resolved in the next three days. He made an uneventful recovery and was closely monitored for features of myocarditis in the second week of illness and neuropathy in the later weeks. Contact tracing for diptheria was carried out, but no probable or possible sources were found. All immediate contacts were initiated on chemoprophylaxis and given a dose of the tetanus-diphtheria (Td) vaccine. On follow up, the boy was vaccinated with a booster dose of the Tdap vaccine.

## Discussion

Diphtheria presents as a pseudomembrane on the site of colonization, usually the tonsils of the patient. There can also be the presence of a sore throat and cervical lymphadenopathy. Rapid administration of diphtheria antitoxin and antibiotic therapy is the mainstay of treatment. Commonly used antibiotics include penicillin and erythromycin, usually for a minimum of two weeks [1]. In India, the National Immunization Schedule (NIS) recommends administration of a pentavalent vaccine (diphtheria, pertussis, tetanus, and hepatitis B and Haemophilus influenzae type b) at six, 10 & 14 weeks of age followed by booster doses of DPT vaccine at 16-24 weeks, and five to six years of age [6].

In our case, the membrane over the tonsils and his partially immunized status led to the suspicion of diphtheria even though Kerala is not a known endemic area. The relatively indolent nature and confinement of the infection to a local area were attributed to the three doses of pentavalent vaccine that the boy had received as part of the NIS. However, the incidence of a co-infection with EBV could have been easily missed. The classical clinical triad of IMN consists of fever, pharyngitis, and lymphadenopathy. Treatment is generally supportive unless complications such as airway obstruction or splenic rupture occurs [2]. The persistence of symptoms despite the administration of antitoxin as well as antibiotics coupled with the deranged liver functions led to the workup and further identification of the EBV co-infection in our case.

The development of a skin rash following the administration of amoxicillin in patients with IMN is quite frequently reported [7]. Since penicillin is recommended in the treatment of diphtheria, in the event of a co-infection with EBV, the use of penicillin can result in the development of such a rash, which can cause further distress to the patient. This makes the identification of such co-infections even more important. 

An extensive review of the literature showed that the incidence of diphtheria and IMN co-infection is a relatively rare clinical entity. Mattos-Guaraldi et al described a similar case in Brazil [3]. Two other cases reported were from the United Kingdom by Perkins et al and Canada by Haight et al [4,5]. The reported cases highlighted the partially immunized status of the patients. All cases had a good clinical outcome following standard treatment of diphtheria similar to ours. 

## Conclusions

We report here a case of diphtheria and IMN co-infection in a partially immunized child from Kerala, India. This case serves to highlight the difficulty in the diagnosis of diphtheria, especially in a non-endemic region. Even if the area is not an endemic one, an inadequate immunization status should prompt the clinician to actively search for evidence of diphtheria. 

We also wish to highlight the possibility of such co-infections which often mimic one another. A high degree of clinical suspicion is warranted in similar cases to avoid missing the second infection. Diphtheria may be mistaken for IMN and vice versa, which can lead to a delay in the correct diagnosis. This may also result in the inadequate treatment of either infection. In the case of such a co-infection, antibiotics such as ampicillin or amoxicillin may be avoided to prevent the IMN rash which can further cause unnecessary anxiety for the patient.
